# Role of Patient-Derived Models of Cancer in Translational Oncology

**DOI:** 10.3390/cancers15010139

**Published:** 2022-12-26

**Authors:** K. F. Idrisova, H.-U. Simon, M. O. Gomzikova

**Affiliations:** 1 Laboratory of Molecular Immunology, Institute of Fundamental Medicine and Biology, Kazan Federal University, 420008 Kazan, Russia; 2 Institute of Pharmacology, University of Bern, 3010 Bern, Switzerland; 3 Department of Clinical Immunology and Allergology, Sechenov University, 119991 Moscow, Russia; 4 Institute of Biochemistry, Brandenburg Medical School, 16816 Neuruppin, Germany; 5 Laboratory of Intercellular Communication, Institute of Fundamental Medicine and Biology, Kazan Federal University, 420008 Kazan, Russia

**Keywords:** patient-derived models, patient-derived xenografts, patient-derived organoids, clinical investigation, 2D and 3D culture, personalized medicine, spheroids, tumoroids, zebrafish patient-derived xenografts, chick chorioallantoic membrane patient-derived xenografts, xenograft-derived organoids

## Abstract

**Simple Summary:**

It was found that long-established tumor-derived cell lines do not adequately reproduce drug sensitivity and behavior of a real human cancers. Therefore, more reliable tumor models that recapitulate the heterogeneity and patho-physiology of patient tumors are under development. The aim of our review is to describe the current patient-derived models of cancer, discuss their advantages and disadvantages, and provide information about clinical trials and inventions in this field. This work provides a comprehensive overview of the strong and weak sides of patient-derived models, as well as the latest achievements in data collection, creation of repositories and biobanks.

**Abstract:**

Cancer is a heterogeneous disease. Each individual tumor is unique and characterized by structural, cellular, genetic and molecular features. Therefore, patient-derived cancer models are indispensable tools in cancer research and have been actively introduced into the healthcare system. For instance, patient-derived models provide a good reproducibility of susceptibility and resistance of cancer cells against drugs, allowing personalized therapy for patients. In this article, we review the advantages and disadvantages of the following patient-derived models of cancer: (1) PDC—patient-derived cell culture, (2) PDS—patient-derived spheroids and PDO—patient-derived organoids, (3) PDTSC—patient-derived tissue slice cultures, (4) PDX—patient-derived xenografts, humanized PDX, as well as PDXC—PDX-derived cell cultures and PDXO—PDX-derived organoids. We also provide an overview of current clinical investigations and new developments in the area of patient-derived cancer models. Moreover, attention is paid to databases of patient-derived cancer models, which are collected in specialized repositories. We believe that the widespread use of patient-derived cancer models will improve our knowledge in cancer cell biology and contribute to the development of more effective personalized cancer treatment strategies.

## 1. Introduction

According to the prognosis of the American Cancer Society, the estimated number of new cancer cases and deaths in 2022 is as follows: 1,918,030 new cancer cases and 609,360 cancer deaths [1]. Cancer incidences continue to increase each year.

Basic and preclinical cancer research have for many years been based on the use of 60 established commercially available cell lines, originally derived from patient samples. Based on these 60 different human tumor cell lines, the National Cancer Institute (NCI), in 1990, created the cancer cells panel NCI-60 which was used for screening of 3000 small molecules per year for potential anticancer activity. The NCI 60 cell line screening panel includes leukemia, melanoma, non-small-cell lung, colon, nervous system, ovary, breast, prostate and kidney cancers [2].

Due to the relative ease of handling and availability, cell lines are widely used in numerous cancer studies. However, it was shown that cancer cells from NCI-60 panel behavior did not closely correlate with corresponding human cancers (ClinicalTrials.gov Identifier NCT02646228). Moreover, some cancer cell lines have been contaminated with other lines [3,4]. Therefore, NCI canceled the protocol of conducting studies using NCI-60 cell lines panel after 25 years of use due to their genetic changes, and as a result of behavioral changes that no longer reflected the behavior of primary cancer cells and, hence, could not serve as a reliable model of cancer cells [5].

Moreover, it was known that cell lines as a tumor model have important limitations, such as lack of interactions with other cell types, lack of influence of cytokines and other cell signaling molecules and loss of tumor tissue architecture [6]. Therefore, more reliable tumor models that recapitulate the heterogeneity and pathophysiology of patient tumors were required. National Cancer Institute decided to replace the NCI-60 with patient-derived xenografts (PDXs) for drug screening [7]. PDX is the most reliable tumor model, which, however, has limitations, primarily related to cost, time, labor and the murine immune system, which cannot replicate the tumor–immune interactions in humans [8].

As a result, other more high-throughput and low-cost models also gained attention and development [9]. Currently, patient-derived models of cancer include maintenance of tumor cells under 2D tissue culture conditions in vitro for a short period of time (primary patient-derived cancer cell cultures—PDC), obtaining three-dimensional structures from patient-derived cancer cells (patient-derived spheroids—PDS, patient-derived organoids—PDO), preparation of patient-derived tissue slice culture (PDTSC), expanding fresh tumor tissues in experimental animals (patient-derived xenografts—PDX), as well as obtaining PDX-derived cell lines (PDXC) and PDX-derived organoids (PDXO). Each of the models has its own advantages and challenges which we will discuss below. Although historically, cell lines were substituted with PDXs as the most reliable tumor model, in our review, all available patient-derived models are discussed based on their complexity and recapitulation of the tumor properties—from the simplest to the most accurate.

Accumulating data of cancer research is collecting and synthesizing in databases. CellMiner (http://discover.nci.nih.gov/cellminer, accessed on 3 December 2022) is a platform that brings together tools for analyzing drug activity, gene expression, and miRNA expression, and was initially built based on NCI-60 experiments. Later, pharmacogenomics datasets such as Connectivity Map [10], the Sanger/Massachusetts General Hospital Genomics of Drug Sensitivity in Cancer (GDSC) [11], the Broad/Novartis Cancer Cell Line Encyclopedia (CCLE) [12], the Broad Cancer Therapeutics Response Portal (CTRP) [13] were created. Data accumulated in these databases provide a comprehensive characteristic of the cancer cell lines that would help to reveal connections between distinct pharmacologic vulnerabilities to characteristic genetic, gene expression, and cell lineage patterns (https://sites.broadinstitute.org/ccle/, accessed on 3 December 2022). Transition from cell lines to patient-derived models stimulated creation of repositories/biobanks of PDC, PDO and PDX, updating the databases with data obtained from patient-derived models and development of computational models of cancer cells.

This work aims to improve our understanding of various patient-derived cancer models by reviewing the latest research, patents and clinical trials in this field, as well as to discuss the benefits and limitations of patient-derived models. Additionally, this review summarizes the latest achievements in data collection and computational analysis in cancer research.

## 2. Patient-Derived Tumor Cell Cultures (PDC)

**Figure 1:**
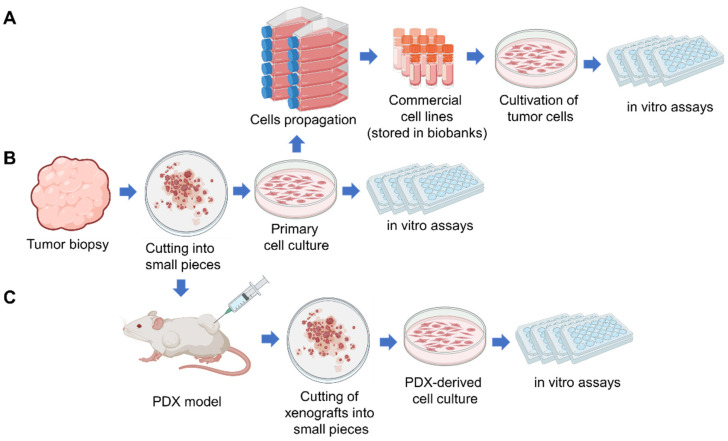
Scheme describing types of tumor cell cultures used in cancer research and procedure of their establishing: (**A**) commercial tumor cell lines, (**B**) primary patient-derived tumor cell culture (PDC), (**C**) PDX-derived tumor cell culture (PDXC). Created in BioRender.com.

Patient-derived tumor cell culture (PDC) is a primary cell culture established from the patient cancer cells which were isolated directly from the tumor tissue or the patient body fluids (ascitic fluid, broncho-alveolar lavage fluid, peripheral blood) (Figure 1) [14,15]. Commonly known cancer cell lines initially were primary patient-derived tumor cell cultures which, as a result of a continuous passaging over a long period of time or immortalization, became widely used cancer cell lines (Figure 1A). Therefore, we can say that the first patient-derived tumor cell culture (PDC) was obtained in 1951, when HeLa cells were successfully cultured in vitro [16]. Currently, HeLa is the oldest and most commonly used human cell line [17].

Human cancer cell lines continue to play a critical role in modern cancer research. Comprehensive characterization confirmed that several hundred cell lines conserve the genomic diversity and the characteristics of the tumor of origin and, consequently, can be used as in vitro cancer models [6,18]. Cell lines are accumulated and stored in biobanks (such as the American Type Culture Collection—ATCC, European Collection of Authenticated Cell Cultures—ECACC, Leibniz Institute Collection of Microorganisms and Cell Cultures—DSMZ, Korean Cell Line Bank—KCLB, Japanese Collection of Research Bioresources Cell Bank—JCRB, Riken Cell Bank—RCD, CellBank Australia), from where cell lines can be distributed and used for research, in preclinical and clinical trials (Figure 1A) [19].

It was shown that some cell lines did not adequately reproduce drug sensitivity and behavior of a real tumor [5,18]. Cell lines have undergone a countless number of cell divisions, acquired mutations due to the long and extensive history of culturing in vitro and are less preferred as a relevant tumor model. Therefore, primary PDCs are gaining increased attention since they are natural replacement for traditional cancer cell lines and reflect the patient’s tumor characteristics and clinical response.

To establish primary PDC, tumor specimens (resection material, biopsies) are first minced into small pieces (1–3 mm^3^), which are subjected to disorganization for single cell suspension by fermental treatment or using various automatic dissociators. Additionally, PDC can be established by isolation of cancer cells from body fluids (Figure 1B). The work of Kim et al. serves as an example of a successful clinical application of the PDC model. The authors generated PDCs from 77 patients with advanced lung adenocarcinoma with a success rate of 24% [20]. It was shown that PDCs maintain patient driver mutations. The authors evaluated the efficacy of anticancer agents as single and in combinations using PDCs in vitro and suggested novel combinational anticancer therapies [20].

However, unlike cell lines which are immortal and easy to culture, primary PDCs are difficult to establish [6]. In order to optimize, scale and improve the success rate of PDCs establishing, the researchers proposed new approaches. In patent KR20180000991(A), a method of PDCs production with subsequent creation of PDX models was suggested. The inventors demonstrated a 73.6% success rate of PDCs establishing from 1 L to 5 L of malignant ascites samples. Afterward, obtained PDCs were injected into mice and were able to form tumors 25–85 days after injection (KR20180000991(A)). It was suggested (WO2022039219 (A1)) to use a conditioned medium from the culture of human neonatal-derived skin fibroblasts and human umbilical vein endothelial cells for PDCs cultivation. This method allowed an increase of efficiency in PDCs establishing compared to the conventional method of cultivation (WO2022039219 (A1)). In patent KR20170127768 (A), a device (anticancer drug screening system) was suggested for establishing PDCs from blood, pleural fluid, ascites, pericardial fluid, amniotic fluid, joint fluid, urine, and cerebrospinal fluid samples, with subsequent PDCs cultivation and comprehensive analysis (KR20170127768 (A)).

Before the PDC as a personalized test system can be introduced in clinical practice, it has to undergo clinical trials. According to the database clinicaltrials.gov (accessed on 30 April 2022), Samsung Medical Center (Seoul, Republic of Korea) tests drug susceptibility of cancer cells in vitro using the PDC model and compares the results with the real clinical outcomes (NCT02646228). National University Hospital in Singapore develops new models of preclinical drug evaluation using cell cultures and xenografts (NCT01130571).

Additionally, PDCs can be established through the expansion of tumor cells in patient-derived xenograft (PDX) models with subsequent tumor cells isolation and seeding in a culture dish (Figure 1C) [7]. PDX-derived cell cultures (PDXC) are useful for high-throughput drug screening and for preliminary analysis before the follow-up of PDX studies [7]. PDX and PDXC are discussed in Section 6 below.

From comparing analysis of different types of tumor cell cultures, it can be concluded that commercial tumor cell lines are the most useful for culturing, and easier and faster to grow in high quantities in short period of time. Whereas primary PDC and PDXC are difficult to establish due to a low success rate and slow growth [21]. The disadvantages of commercial tumor cell lines are low cells heterogeneity, absence of stromal and immune components and significant deviation in properties from the original tumor observed for some cell lines [18]. At the same time, primary PDC and PDXC are characterized by tumor cell genetic and differentiation heterogeneity and presence of tumor-associated cells that more closely resemble the original tumor [18]. In general, 2D tumor cell culture is characterized by a comparatively short period of obtaining high-throughput screening opportunities and low cost. However, the main limitation of PDCs is that 2D culture does not fully reproduce the tumor architecture [18,21].

## 3. Patient-Derived Spheroids (PDS) and Organoids (PDO)

Tumors are three-dimensional (3D) formations experiencing heterogeneous oxygen and nutrients availability, metabolite removal and drugs exposure. As a result of oxygen depletion and insufficient vascularization in solid tumors, hypoxic regions are developed, that induce alterations in tumor cells metabolism [22]. Therefore, to model tumor structure in vitro as close as possible to in vivo conditions, 3D culture models in the form of spheroids and organoids have been developed [22].

Spheroids are aggregates of cells which were obtained by direct cell concentration and cultivation in a small medium volume. Spheroids cannot self-assemble and do not require a scaffold. In other words, spheroids are layers of cells temporarily assembled by intercellular adhesion into three-dimensional cellular structures. Sutherland et al. first described spheroids in 1971 [23]. The authors grew V79 Chinese hamster lung cells in suspension culture, resulting in the formation of the multicellular spheroids, which contained weakly cycling cells and morphologically had the properties of some animal and human carcinomas [23]. Then, tumor spheroids were obtained from brain, breast, lung, colon, prostate, pancreas, and ovarian tumors [24,25,26]. The term “tumorspheres” is used in the literature to underline the cell source used for the production of spheroids [26]. Tumorspheres are a mixture of tumor cells and cancer stem/progenitor cells, and can survive and proliferate without attachment to the surface [27].

Organoids are initially more complex in histological and genetic structure and resemble the original tumor which they originated from [24,28]. Organoids are formed by stem, progenitor cells or tumor cells with stemness properties and recapitulate the phenotypic, genetic, and transcriptomic characteristics of the original tissue or organ. The distinguishing feature of organoids over spheroids is their ability for self-organization of cells on a scaffold, involving differentiation of cells forming a complex tissue structure [29]. The main advantage of organoids is recapitulating primary tissue structure (which often possesses a central lumen) and function by different cell lineages, at least in part [24,30] [https://www.atcc.org/, accessed on 3 December 2022].

Organoids originated from tumor cells called “tumoroids”, are histologically similar to the original tumor, easy to propagate and are suitable for high-throughput systems [31,32]. The first organoid was successfully obtained by Hans Klevers in 2009 using LGR5 1 stem cells isolated from the bottom of the crypts of the small intestine [33]. To date, cancer organoids have been developed from many types of cancer, including stomach cancer [34], colorectal cancer [35], liver cancer [36], pancreatic ductal adenocarcinoma [37], prostate cancer [38] and breast cancer [39].

Spheroids and organoids are different 3D cell culture models that are simultaneously applied in cancer research to achieve higher throughput and reliability. The first step of spheroids and organoids production is mechanical or enzymatic dissociation of tumor tissues or isolation of cancer cells from body fluid (ascitic fluid, urine, broncho-alveolar lavage fluid, peripheral blood). The second step is cultivation of isolated cells; it differs for spheroids and organoids and is described below.

Patient-derived spheroids (PDS) are created using suspension of individual cells obtained by mechanical or enzymatic dissociation of tumor tissue or detachment of cells from culture dish (if cell line is used to generate spheroids). The process of spheroid formation requires a method promoting cell-to-cell interactions that leads to aggregation of cells, namely: (1) liquid overlay technique (LOT); (2) hanging drop; (3) suspension culture based on agitation or magnetic levitation; (4) micromolding microwells; (5) scaffold-based, (6) immersion bioprinting (Table 1).

PDS as an in vitro tumor model has a number of advantages: they reflect a three-dimensional tumor architecture, have a low price of establishing and are suitable for large-scale screening. The disadvantages of PDS are poor structural organization and lack of different cell lineages. A more advanced model that can maintain the heterogeneity of original cancers and can recapitulate the tissue structure is the organoid model [8]. Therefore, most in vitro research is currently shifting to the use of organoids as a model that more closely resembles the original tumor tissue.

Patient-derived organoids (PDOs) are created by cultivation of isolated adult cancer cells with stemness properties in the ECM scaffold (basement membrane extract, Matrigel or Geltrex) covered by culture medium [55]. First, organoids from adult stem cells of intestine epithelium were obtained by Sato and colleagues [33]. The authors used the culture medium containing growth factors stimulating epithelial cells proliferation and differentiation: an activator of Wnt signaling pathways (R-spondin ligand); a ligand of tyrosine receptor kinases (epidermal growth factor (EGF)), an inhibitor of transforming growth factor-β/bone morphogenetic protein signaling (Noggin) [33]. It appeared to be a hard task to grow tumor organoids due to a higher rate of proliferation and overgrowth of normal epithelium. Van de Wetering and colleagues reached a ~90% success rate of growing patient-derived colorectal cancer organoids by removing Wnt activators from the culture medium [56]. Cancer cells often carry mutations that activate the Wnt pathway whereas normal cells require external addition of Wnt signaling pathways activators for their growth [55], therefore, removal of Wnt from the culture medium prevented the growth of normal epithelium and increased the success rate of organoids production. Analysis of obtained organoids by H&E staining, marker expression analysis (KI67, OLFM4, KRT 20, Alcian blue) revealed heterogeneity between patients and within individual organoids of each colorectal cancer patient [56].

Organoids and spheroids represent a more reliable in vitro cancer model. It was found that, compared to 2D cell culture, tumor spheroids are fairly resistant to anticancer drugs as well as radiation [57,58]. Numerous studies have shown a positive correlation between the in vitro response of organoids to drugs and corresponding response of primary tumors in vivo in both mice and humans [34,36,59]. Therefore, many of the clinical trials using organoids were initiated according to the database clinicaltrials.gov (accessed on 30 April 2022). For example, in the University Hospital Inselspital in Berne, patient derived organoids (PDO) are formed from the biopsies of bladder cancer (33 participants) and used for drug screening and identification of suitable treatment for patients (Phase 2, NCT05024734). Joint scientific group of several institutions in the USA and Canada are investigating the concordance between PDO chemotherapy sensitivity and response in patients with metastatic pancreatic ductal adenocarcinoma (150 participants) (Phase 2, NCT04469556). The PDOs predictive ability of drugs efficacy is evaluating in non-small cell lung cancer (NCT03453307), lung cancer (NCT03979170) and colon cancer (NCT04906733). The University of Texas Health Science Center at San Antonio is creating a live biobank of PDO from patients with stage I–IV lung cancer (NCT03655015).

Depending on the protocol of organoid culture establishment, organoids can be formed from a single cell type (cancer stem cells) or using several cell types (cancer and stromal cells). The limitation of PDO formed from only CSCs is absence of immune and stromal non-immune components (mesenchyme, endothelial, nervous and muscle cells) of the tumor microenvironment, which significantly affect the survival of tumor cells [60,61]. Therefore, to more completely recapitulate the TME, a 3D co-cultivation approach of organoids with tumor associated cells is applied [8], which is discussed in the following section.

## 4. Complex PDO Model

The tumor microenvironment includes extracellular matrix (ECM) and stromal cells such as cancer-associated fibroblasts (CAF), immune cells (granulocytes, lymphocytes, macrophages), which play a critical role in tumor development and drug resistance. To model direct cell-to-cell communication that takes place in a tumor but under in vitro conditions, co-cultivation of organoids with tumor-associated cells is performed. This approach usually involves growing organoids, cancer-associated fibroblasts and immune cells separately and then creating a complex tumor organoid culture (reconstitution approach) [8]. It should be noted that the protocol for organoid establishing can be modified so that small fragments of tumor tissue were preserved and intratumoral stromal cells were included in the forming complex organoids (holistic approach) [62].

One of the key components of the tumor stroma is CAF—cancer associated fibroblasts, that can be derived from resident fibroblasts, epithelial cells, endothelial cells (via epithelial–mesenchymal transitions—EMT), migrated bone marrow-derived and adipose-derived mesenchymal stem cells (MSCs) [63,64,65,66,67,68], stellate cells [69,70]. CAF create and remodel the structure of the extracellular matrix (ECM) [71], affect angiogenesis, drug access and response to therapy, modulate activity of immune cells [72]. CAF-derived factors can induce a tumor-supporting microenvironment and promote cancer cell metastasis [73]. Therefore, 3D co-culture models of tumor organoids with CAFs were established.

Öhlund et al. developed in vitro model of intercellular communication between CAFs and tumor organoids obtained from pancreatic ductal adenocarcinoma. To model direct cell-to-cell contact, the authors digested tumor organoids into single cells and seeded them together with CAFs into a culture plate [74]. To model paracrine signaling in tumor microenvironment, the authors used a transwell system, where CAF cells were seeded in the upper compartment of transwell and organoid-derived single cells were seeded in the lower compartment [74,75]. Later, Liu et al. applied the described methods to investigate the role of intercellular communication between mouse or human liver tumor organoids and CAFs. The authors found that CAFs promote tumor organoid growth and increase resistance to anticancer drugs in direct co-culture and in a transwell system [75].

Tsai et al. created co-culture model consisting of three types of cells: tumor cells, CAFs and T-lymphocytes [76]. The authors cultured organoids from the tumor tissues of 28 patients with pancreatic cancer (including primary and metastatic tumors, ascites, rapid autopsy specimens) and found that initial organoid cultures included a mixed population of epithelioid organoids and fibroblasts [76]. These cancer-associated fibroblasts that attached to the well surface were isolated from organoids. Human T-cells were isolated from human frozen blood buffy coats [76]. Next, the authors plated organoids and patient-matched CAFs in Matrigel and added T-lymphocytes in the organoid growth media. It was found that T-lymphocytes infiltrated the Matrigel, migrating toward the organoids. Importantly that co-culture of organoids with stromal cellular components (CAF and infiltrated T-lymphocytes) increased drug resistance of cancer cells [76].

Chakrabarti et al. generated an autologous organoid/immune cell co-culture system using gastric cancer organoids, bone marrow-derived dendritic cells (DCs) and cytotoxic T-lymphocytes (CTLs) [77]. The co-cultivation system revealed that DCs present tumor-specific antigens to CTLs and induce PD-1 expression on these lymphocytes, at the same time tumor cells express inhibitor of CTL function—programmed cell death ligand 1 (PD-L1) that leads to protection of cancer cells from anti-tumor lymphocyte activity [77]. Co-cultures of autologous tumor organoids and peripheral blood lymphocytes for individual patients were described by Dijkstra et al. [78]. Tumor organoids were dissociated into single cells and added to the PBMC culture. The authors proposed to use this co-culture system with tumor organoids to expand patient-specific tumor-reactive T cells as an anticancer adoptive cell therapy [78].

The advantage of this co-cultivation method is a closer resembling of tumor tissue. The disadvantage is a limited reproduction of intercellular and cell–matrix contacts, incorrect ratio of cell types and labor intensity.

In contrast to building TME in tumor organoids by co-culturing few cell types, it was suggested to seed patient tumor samples directly into Matrigel without a tumor tissue dissociation step. This procedure allows to preserve tumor cell–matrix interactions and stroma cells heterogeneity [79]. In this method, tumor tissues are cut into fragments, which are embedded in a collagen gel and placed in the upper compartment of a transwell. The lower compartment of the transwell is filled with a culture medium, whereas the top of collagen embedded tumor tissue is exposed to air, hence, the method is called air–liquid interface (ALI) culture. In the ALI method, organoids grow without the addition of external factors, since these factors are believed to be produced by stromal cells within the organoids [80]. This approach slightly resembles the method of patient-derived tissue slice culture, which is discussed in more detail below.

## 5. Patient-Derived Tissue Slice Culture

Tissue slice culture (TSC) is well known technique used to study the physiologic processes of normal tissues, for example lung tissue [81], nervous tissue [82]. The method of TSC consists in placing tissue or organ in 1.5% agarose, then cutting of agarose blocks using a vibrating microtome and cultivating of obtained slices in culture medium. The method gained renewed attention when it was applied to tumor tissue.

The patient-derived tissue slice culture method consists of taking tumor tissue using a sterile biopsy punch, cutting about 200–500 µm thick tumor slices using a vibrating microtome, placing the obtained PDTSCs in culture plates containing growth media and incubation of PDTSCs under shaking [83]. To increase preservation and survival of cells, PDTSCs can be cultured on membrane inserts [84], which provide a mechanical support for the tumor slices, and increased oxygen supply compensated the lack of a functional blood supply in PDTSC.

Carranza-Torres et al. also showed that metabolic activity and morphological integrity of breast cancer PDTSC were conserved for at least 72 h [85]. Zech et al. found that PDTSC of oropharyngeal squamous cell carcinoma maintained stable oxygenation and proliferation characteristics for at least 3 days ex vivo. Using this model, the authors were able to detect DNA double-strand break (DSB) repair deficiency in cancer cells [86]. Longer retention of 3D architecture, cell viability, pathway activity, and global gene-expression profiles in PDTSC for up to 5 days culturing ex vivo were demonstrated by Vaira et al. [87]. It was shown that T cells and macrophages were able to survive in PDTSC cultured for over a week [88]. Van De Merbel et al. developed an improved ex vivo culture method by culturing PDTSC on filter inserts (with pore size of 4 μm) under hyperoxic (95% O_2_) conditions. Using this model, the authors found that PDTSC from human prostate and bladder tumors can be cultured without detectable quality loss (cells remained viable, the tissue structure was preserved) for up to 5 days. After 10 days of ex vivo culture, nuclear fragmentation and protein degradation in cells were observed that are indicatives of gradual loss of tissue architecture [89].

Kenerson et al. confirmed that PDTSCs reflect the metabolic activities, tumor cell density, proliferation rate, heterogeneity and clinical response to chemotherapies of the tumor [83]. In this context, PDTSC is a suitable alternative model for evaluating effectiveness of anticancer compounds in a complex tumor microenvironment [85,90].

PDTSC allows to investigate the influence of anticancer drugs on activity and viability of tumor associated cells. Stenzel et al. have shown that treatment of renal cell carcinoma PDTSC with nivolumab led to significant reduction of programmed death receptor-1 (PD-1) expression, as well as effected density and proliferation of tumor-infiltrating CD8+ T cells [91].

Importantly, automated PDTSC analysis can be established to select and prescribe the most promising drugs for individual patients. Sönnichsen et al. described an automatized pixel-based readout applied to manually taken fluorescent images of PDTSC of colorectal carcinoma specimens [92]. The authors were able to obtain reproducible data of proliferative activity of tumor cells [92]. Martin et al. applied an automated readout of proliferation and tumor-morphometry to PDTSC for quantification of drug susceptibility of metastatic colorectal cancer [92].

The advantages of patient-derived tissue slice culture (PDTSC) as a 3D cancer model ex vivo are preserving histopathology, tumor microenvironment, including intercellular contacts and a wide variety of cells. An additional advantage of PDTSC compared to PDO obtained by the ALI method is that it is less time consuming due to fewer manipulations. PDTSC model can be established within a day; study of PDTSC response to chemotherapies takes about 2 days (depending on treatment period) [84]. The disadvantages are applicability to solid malignancies only [85], do not completely reflect the complexity of a tumor and have a finite lifespan. The advantages and disadvantages of PDTSC are summarized in Table 2.

## 6. Patient-Derived Xenografts

Much emphasis has been placed on patient-derived xenografts (PDXs) as the most reliable model for the replacement of the NCI-60 panel. The PDX model is created by direct transplantation of part of a freshly excised patient tumor into an animal model. Initially, PDXs were established in mice and a mouse PDX model (mPDX) is still the gold standard in oncology. Due to the limitations of an mPDX model, alternative PDX and PDX-derived models were developed such as zebrafish patient-derived xenografts (zPDXs), chick chorioallantoic membrane patient-derived xenografts (CAM-PDXs), humanized mPDX, PDX-derived organoids (PDXO) and cell cultures (PDXC).

### 6.1. Mouse Patient-Derived Xenografts (mPDXs)

The procedure of PDXs establishing consist of collecting part of human primary tumor tissue or metastasis tissue from postoperative material or biopsy. Tumor samples are placed in a medium with antibiotics and transported to the laboratory at 4 °C. Then, the tissue is cut in 3 × 3 × 3 mm^3^ fragments and one tumor fragment per mouse implanted subcutaneously by making a superficial incision in the skin [93]. Through about 3–6 months, PDX tumors are formed. When the PDX tumor reaches 1 cm^3^ in size, it is isolated and fragmented for the subsequent cryoconservation and biobanking, as well as histological, molecular characterization or passaging of tumor material from animal to animal [94]. The first generation is called F1 and the subsequent generations are named F2, F3.

After isolation, PDXs can be stored in cryostorage. PDXs are sliced into 5 × 2 mm pieces, then 1–2 slices are put in a vial preloaded with cryopreservation media (containing 10% Dimethyl sulfoxide (DMSO) with 10% fetal bovine serum in medium) or commercial specialized cryoprotectant [94].

PDX can be established using patient samples of ascitic fluid [95] or pleural effusion [96]. Tumor cells are isolated from the samples by centrifugation, mixed with Matrigel and injected subcutaneously in immunodeficient mice [97]. The tumor tissue is more frequently implanted subcutaneously or orthotopically. Subcutaneous transplantation is the simplest method for implementation and assessment of tumor growth that does not require special equipment. Orthotopic transplantation is engraftment into the anatomically appropriate site corresponding to the cancerous tissue origin. Orthotopic transplantation better reproduces the tumor microenvironment [98]. In addition to the subcutaneous and orthotopic transplantation, tumor tissue can be transplanted into the intracapsular fat pad, the anterior compartment of the eye, under the renal capsule [99] into the spleen [7] and intramuscularly [99].

Kamili et al. compared the efficiency of PDX establishing using primary and metastatic solid tumor samples, as well as subcutaneous, intramuscular and orthotopic methods of engraftment [100]. Interestingly, PDX models were established for 44% of patients at diagnosis and 100% at relapse. It has been previously noted that successful tumor engraftment in PDX models is correlated with poorer prognosis for patients [101]. Orthotopic engraftment was more effective than subcutaneous or intramuscular engraftment [100].

It was shown that the rate of exponential PDX tumor growth is increasing with in vivo passage [102]. Genetic stability of the PDX model during in vivo mouse-to-mouse serial passages has been questioned [103]. Recent work has shown the genomic accuracy of PDX models with respect to patients’ original tumors [104]. PDX models have proven to be highly predictive in biomarker detection and drug testing for both molecular compounds and chemotherapy [105,106,107].

Advantages of mPDX are recapitulation of complex tumor microenvironment, tumor cell heterogeneity and original architecture of the tumor, immunomodulation and exposure of tumor cells to a wide spectrum of cytokines, chemokines, growth factors and hormones in vivo (Table 2) [108]. Mouse models of PDX, widely used in cancer research, have made enormous contributions to our understanding of the biology of cancer. However, PDX models in mammals have a few disadvantages. Firstly, it takes 4 to 8 months to create a complete PDX cohort for in vivo drug screening (Table 2). Whereas most patients, for example, having refractory cancer, live less than 1 year (NCT02646228) and are less likely to receive screening results. Secondly, depending on the type of tumor, the rates of successful engraftment and growth rates of implanted tumors range from 25–75%. Next, maintaining PDX mouse models is laborious, time consuming and expensive.

### 6.2. Zebrafish Patient-Derived Xenografts (zPDXs)

An alternative PDX model is a zebrafish patient-derived xenograft (zPDX) model which is widely applied in cancer research due to low-cost, high-throughput and quick establishment. Although zebrafish are not mammals and differ in body temperatures, they have musculoskeletal, cardiovascular systems, eyes, brain, liver, heart, gastrointestinal tract and pancreas similar to those of mammals [109].

The procedure of zPDX establishing consists of anesthetizing 2 day old zebrafish embryos and injecting tumor cells into the embryos using a microinjector [110]. The most commonly used sites of tumor cells injections are the yolk sac, perivitelline space (space between the yolk sac and the periderm) or duct of cuvier (common cardinal vein) [111]. At 24–96 h postinjection, the zPDX model can be imaged and analyzed for transplanted cancer cell proliferation and migration [110].

Transparent and translucent features of zebrafish make them an ideal model for investigating the development of tumor vasculature, tumor growth and metastasis [109]. To visualize blood vessels and investigate tumor-induced angiogenesis, genetically modified zebrafish with vascular endothelial cell-specific expression of GFP have been generated. Two of the most commonly used lines are called fli1a:EGFP [112,113] and used to study tumor neovascularization and evaluate the efficacy of antiangiogenic agents [109,114]. In addition, fluorescent staining of cancer cells before the injection into GFP-expressing zebrafish allows investigation of intravasation and extravasation of cancer cells. Importantly, that monitoring of cancer cell behavior can be done non-invasively within the host using standard epifluorescence and confocal microscopes [114]. For example, cancer cells (Hs578T, MDA-MB-468, SW620, HT29, HCT116, as well as cells isolated from breast cancer and colorectal cancer surgical resected samples) were stained with lipophilic dyes CM-DiI or vital dye Deep Red and injected into fli1:eGFP zebrafish to study tumor-induced angiogenesis and evaluate the effectiveness to anti-VEGF therapy using bevacizumab. The authors showed that zPDX model reflects the clinical effects under bevacizumab treatment of patients [115]. Di Franco et al. demonstrated that a concordance between the response of the patient to chemotherapy and the outcomes reported in the corresponding zPDX was registered in 75% of metastatic colorectal cancer patients [116].

It was found that relative sensitivities to chemotherapeutic drugs determined using zebrafish (zPDX model) are maintained in the rodent model (mPDX) [117]. The unique advantages of zPDX as an animal model are rapid turnaround, low-cost and high-throughput, since zebrafish can hatch 150–200 eggs every week. Quick establishing of zPDXs and obtaining results (about 72–144 h) make them suitable for short-term clinical treatment. In addition, the zPDX model is characterized by small space requirements and low doses of screened drugs (Table 2) [109,118]. In contrast to the mPDX model, zPDX has small sample size requirements (hundreds of cells are enough for transplantation) and require relatively minimal maintenance and care [114].

Among the shortcomings of zPDX are high mortality after xenograft injection, difficult translatability of results obtained by dosing in water, complicated administration of hydrophobic drugs and intravenous injection, as well as different body temperatures between fish and humans (Table 2) [109]. Zebrafishes prefer an environmental temperature of 28 °C; however, they are capable of surviving at temperatures from 32 to 36 °C, closer to human cell culture conditions [114]. After tumor cells transplantation, zebrafish embryos are usually incubated at 33 °C as a compromise allowing growth of both human cancer cells and fish [110]. However, human cells housed in slightly cooler temperatures have a significantly altered metabolism that results in reduction of proliferation, changes in survival, and response to chemotherapy [118].

### 6.3. Chick Chorioallantoic Membrane Patient-Derived Xenografts (CAM-PDXs)

The chicken egg chorioallantoic membrane (CAM) is one more alternative in vivo PDX model. Since the chicken embryo lacks a functional adaptive immune system until Day 18 and is considered a non-life animal, the CAM models are more convenient to establish [119,120]. In the fertilized eggs on day 7 or 8, when CAM has fully developed a “window”, they are prepared by drilling the shell and disrupting the inner membrane. Then, a tumor fragment (3 × 3 × 3 mm^3^) is transferred to the CAM near the “Y” bifurcation of a blood vessel and left to grow. The tumor can be formed in 3–4 days after transplanting human tumor samples [119,121,122]. Success of CAM transplantation reaches 73% using fragments of recurrent respiratory papillomatosis tissue [123], 100% survival of transplanted tissue fragments of laryngeal squamous cell carcinoma was observed [124].

The CAM has historically been used to study angiogenesis induced by cancer cells. Its high vascularity stimulates tumor growth and serves as a model for screening of anti-angiogenic agents [119]. After transplantation tumor grafts become vascularized by chick vessels within 2–5 days that supports rapid tumor growth in volume and mass [125].

Analysis of tumor growth on the CAM is usually relied on visual inspection by microscopy and quantification of nodule size or weight. Pawlikowska et al. have shown that metastatic capacity of tumor cells following implantation onto the CAM can be evaluated using 3D live imaging (IVIS Spectrum system) [126].

CAM-PDXs have been shown to maintain the heterogeneity, pathophysiology, and major morphological and cellular features of the original tumors [119,127]. CAM-PDXs are characterized by high successful transplantation rate which reaches to 80–100% for glioblastoma, sarcoma, nasopharyngeal carcinoma, and renal cell carcinoma [125].

The advantages of CAM-PDX model are low-cost, easily-visualized and manipulatable (Table 2). This model allows simultaneous screening of large numbers of pharmacologic agents (high-throughput). Cancer cells can be implanted easily, non-invasively, and in an immunodeficient environment [128]. Establishing of CAM-PDXs and obtaining results take about 2 weeks. However, from another point of view, this advantage (short time to obtain the result) does not obtain visible metastasis due to the short observation period. In addition, the different drug metabolism and immune system in chicks as compared with mammals might lead to distinct drug responses [119].

### 6.4. Humanized Mouse Patient-Derived Xenografts (Humanized mPDX)

Next-generation forms of PDX are humanized models in which mice are injected intravenously with human-derived CD34^+^ hematopoietic stem cells (HSCs) to create a competent human immune system [Stripecke, et al., 2020]. PDXs with a human immune system help to study interactions between human cancer and human immune cells. Successful engraftment of human immune cells assesses by detection of differentiated human CD45^+^ cells in mice peripheral blood [129]. To support proliferation and differentiation of transplanted human immune cells, transgenic and knockout strains have been created that produce growth factors such as IL-3, GM-CSF, SCF, TPO and/or M-CSF, and others [130]. Humanized mPDXs more accurately reflect the disease phenotypes observed in patients and provide more clinically relevant results when used in drug screening. Using humanized mPDX, Morton et al. demonstrated, that human immune cells maintained the microenvironment of engrafted cancer PDXs [129,131]. One of the limitations of humanized PDX models is allogeneity of human immune cells to transplanted tumors, since it is usually difficult to take HSCs from cancer patients. Allogeneic immune cells distort the pattern of interaction between cancer and immune cells in PDX.

### 6.5. PDX-Derived Organoids (PDXO) and PDX-Derived Cell Cultures (PDXC)

**Figure 2:**
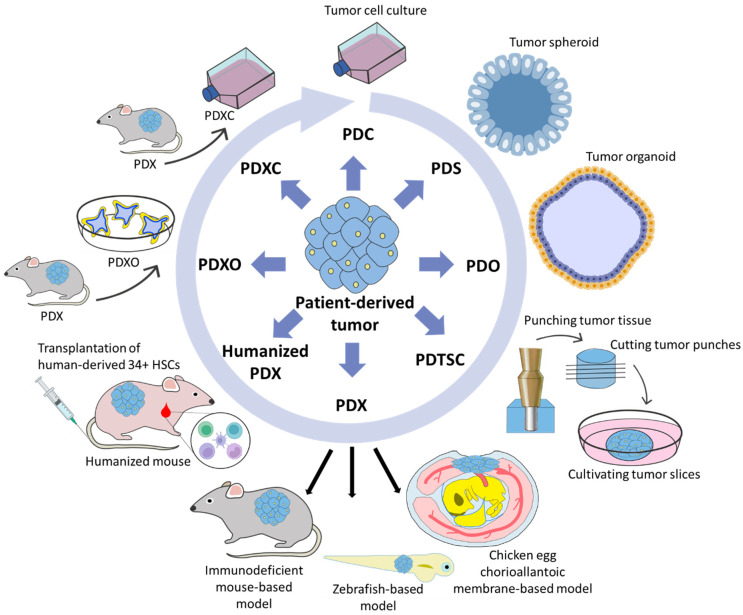
Patient-derived models: PDC—patient-derived cell culture, PDS—patient-derived spheroids, PDO—patient-derived organoids, PDTSC—patient-derived tissue slice culture, PDX—patient-derived xenografts (based on mice, zebrafishes or chicken egg chorioallantoic membranes), humanized PDX, PDXO—PDX-derived organoids, PDXC—PDX-derived cell culture. The described models are shown in order of more complexity and the greatest repetition of a patient tumor. The closed circle represents that the more complex the model and the closer it reproduces the properties of the tumor, the further it is from high-throughput screening, forcing a return to PDX-derived cell organoids and culture which have the greatest high-throughput.

The mouse PDX model has a high cost and requires extended time to conduct experiments, therefore researches generate PDX-derived organoids (PDXO) and primary PDX-derived cell cultures (PDXC) (Figure 2) to obtain a platform for large-scale screening of anticancer drugs.

The procedure of obtaining PDXC consists in isolating a tumor graft from animals with PDXs, cutting it into small pieces followed by disorganization into individual cells (similar to the usual procedure for obtaining a primary cell culture from a tumor biopsy) and seeding on a culture dish (Figure 1). The PDXO establishing procedure consists of mild tumor dissociation followed by differential centrifugation aimed to enrich for organoids and depleted single cells [132].

Huang L. et al. compared PDXO drug response with mPDX in vivo response data and demonstrated the applicability of PDXO to predict response to drugs in vivo [133]. Guillen K.P. et al. revealed consistency between drug screening results in PDXO and drug responses in breast cancer mPDX [132]. Xu et al. compared PDXO and PDXC with corresponding mPDX and found that PDXO and PDXC show model-specific growth kinetics and drug response: the greatest overlap in drug response was observed between mPDX and the corresponding PDXO [134].

The main advantages of PDXC and PDXO over the parental mPDX model are lower cost of establishing and high-throughput. Importantly, PDXC and PDXO have matching PDX models, that allows the conducting of a large-scale screening in the first step of research, select potential drug candidates and return to mPDX model on the second step. This experimental scheme allows the reduction of the number of animals used in the experiment, validation of obtained data using the in vivo mPDX model and better comparability of in vitro and in vivo data [135]. However, these models are characterized by all the above-described disadvantages for cell cultures and organoids, respectively, i.e., absence of original tumor architecture, lack of complex tumor microenvironment, absence of exposure to wide spectrum of cytokines, chemokines, growth factors and hormones.

Summarizing the above, we can conclude that animal PDX is a physiologically relevant tumor model that is able to accurately reproduce tumor cell diversity, tumor progression, including the development of metastases [136,137]. Therefore, PDX models are extensively exploited in clinical trials for the treatment of patients with metastatic renal cell carcinoma (CAM-PDX model, NCT04602702), recurrent metastatic head and neck squamous cell carcinoma (mPDX model, NCT02752932), metastatic prostate cancer (mPDX model, NCT03786848). Another clinical trial (mPDX model, NCT03134027) investigates the generation of humanized mPDX model using human blood CD34+ hematopoietic stem/progenitor cells (HSPCs) to recreate the corresponding human immune system in a PDX model and avoid rejection of tumor engraftment by the host’s immune system. After successful completion of clinical investigations, the PDX method of drug screening can be introduced into clinical practice.

## 7. Databases and Computational Models of Patient-Derived Cancer Models

The National Cancer Institute (NCI) is developing a national repository of patient-derived models (PDM) consisting of about 1000 patient-derived xenografts (PDX), patient-derived tumor cell cultures (PDC), patient-derived organoids (PDO) as well as cancer-associated fibroblasts (CAFs). These PDMs are clinically annotated, have an established growth curve, are molecularly characterized and the information is available in a readily accessible database (information from https://pdmr.cancer.gov/, accessed on 3 December 2022). Moreover, NCI jointed efforts with National Human Genome Research Institute to implement the cancer genomics program—the Cancer Genome Atlas (TCGA), aimed to generate genomic, epigenomic, transcriptomic, and proteomic data of primary cancers spanning 33 cancer types (www.cancer.gov, accessed on 3 December 2022).

EurOPDX is a consortium of 16 European institutions that holds 1500 PDX models [5]. Jackson Lab has 450 PDXs. The Swiss pharmaceutical company Novartis uses 1000 PDX models for drug screening. Trusolino et al. reported about creating and maintaining collection of 600 colorectal cancer PDX [5].

The power of “big data” available in the described resources was shown by Uhlen M. et al. The authors analyzed 7932 tumor samples collected from 17 major human cancer types using database TCGA (The Cancer Genome Atlas) and HPA (Human Protein Atlas). They showed that shorter patient survival was associated with up-regulation of genes involved in cell growth and with down-regulation of genes involved in cellular differentiation. Using genome-scale metabolic models, the authors showed that cancer patients have widespread metabolic heterogeneity, highlighting the need for precise and personalized medicine for cancer treatment [138].

Creation of computer models using biological data obtained from individuals can help in the study of patient response to treatment and development of personalized medicine. There is a repository of mathematical models of biological and biomedical systems called BioModels EMBL-EBI [https://www.ebi.ac.uk/biomodels/, accessed on 3 December 2022], which includes 6753 models derived from tissue samples obtained from cancer patients. Breems et al. used a mathematical model of the interaction between mouse melanoma cells, Th2/Th1 cells, and M2/M1 macrophages to investigate the role of M1 and M2 macrophages polarization in tumor growth. The authors showed that tumor growth is associated with Th2 immune response and M2 cells [139]. Eftimie et al. investigated dynamics of Th1 and Th2 cells, as well as M1 and M2 macrophages in the presence/absence of tumor cells using mathematical models. The authors found that tumor elimination was mainly the effect of M1 macrophages and to a lesser extent the effect of Th1 cells [140]. Shin et al. developed a mechanistic model of the integrated EGFR-PYK2-c-Met signalling network to identify potent drug combinations for the treatment of breast cancer. Their results showed that co-targeting of EGFR and PYK2 and to a lesser extent of EGFR and c-Met yielded the strongest synergistic anti-cancer effect [141]. Li et al. investigated crosstalk between cancer cells and macrophages in the tumor microenvironment using in silico co-culture computational models. The authors included the following parameters: polarization of macrophages (M1 vs. M2), epithelial–mesenchymal plasticity of cancer cells, and interconnection of cancer cells exhibiting different phenotypes (epithelial vs. mesenchymal) with polarization of macrophages. Importantly, the authors found that the system can reach a stable steady state where cancer cells become extinct [142].

## 8. Conclusions

A tumor consists of cells characterized by enormous biologic and genomic heterogeneity. Therefore, the only way to effectively study the behavior of cancer cells and select drugs is to create patient-specific models. Great work has been done in this direction and currently repositories of patient-specific models have been created and a large amount of molecular data has been accumulated, providing significant progress in basic research, drug development and clinical treatment.

Based on the data reviewed, it can be concluded that each patient-derived model has strong and weak sides, pros and cons. So, in line PDC-PDS-PDO-PDTSC-PDX, the complexity and accurately of the models to the original tumor are improving, whereas such metrics as throughput, cost and labor are worsening. It can be concluded that the more complex the model and the closer it reproduces the properties of the tumor, the further it is from high-throughput screening, forcing a return to more throughput PDX-derived cell organoids and culture. This pattern resembles the closed circle. However, this pattern is disturbed by such models as zPDX and CAM-PDX, the strong sides of which are quick establishment, high-throughput and low-cost. Nevertheless, the non-human immune system and short observation period do not allow the solving of all research and clinical tasks only with the use of zPDX and CAM-PDX models. Therefore, mPDX is still the gold standard in oncology. An important function is performed by PDXO and PDXC, which bridge the gap between traditional in vitro studies and in vivo PDX studies [143]. We believe that patient-derived models should be considered complementary, and the model should be selected based on the aims of the study. For example, the first step of drug screening should be carried out using high-throughput models such as cell cultures, spheroids, organoids, as well as PDX-derived cell cultures and organoids, the second step of screening should be based on high-throughput animal models such as zPDX and CAM-PDX, and finally, activity of the most promising candidates should be confirmed using an mPDX model.

## Figures and Tables

**Table 1:** Methods of spheroids obtaining.

Method	Description	Reference
Liquid overlay technique (LOT)	Cell suspension is seeded on a non-adhesive substrate, such as low adhesion or agar-coated plates preferably with round bottom, to prevent cells from attaching to surfaces. Advantages of LOT technique are relative ease of implementation, low price and ability to track spheroids in real time. The disadvantages are inconsistent size and shape of spheroids.	[40]
Hanging drop	Cell suspension (20–40 μL) is dropped onto a cap, which is then turned over, and cell aggregation occurs at the top of the drop due to surface tension and gravity. Advantages of this method are ease of handling and low price. The disadvantages are that it is labor intensive, limited in cultivation time and there are difficulties in observing the spheroids formation.	[41]
Suspension culture based on agitation or magnetic levitation	Cell suspension is cultivated in a rotating flask or bioreactor with agitation or magnetic levitation. Constant stirring prevents cells from setting and attaching to the surfaces of a device, and the use of a medium with increased viscosity stimulates intercellular adhesion. Advantage of this method is large yield of spheroids (~300). The disadvantage is large variation of obtained spheroids in size and not uniformity.	[42]
Micromolding microwells	A recently developed method for obtaining spheroids using arrays of micropits made by microforming or photolithography. Low adhesion surfaces are obtained using non-adhesive materials such as polydimethylsiloxane or by coating with agarose. Advantages of this method are spheroids formation with specific size and composition, small amount of cells, media, and reagents are required. The disadvantages are complexity and high cost of the equipment, as well as the inability to extract and characterize in detail the formed spheroids.	[43]
Scaffold-based	Cells are embedded into the matrix resembling extracellular matrix (ECM) of biological origin (collagen, fibrin or Matrigel) or synthetic (hyaluronic acid (HA), polyethylene glycol (PEG), polylactic acid (PA), polyglycolic acid (PGA)). The advantage of this method is replicating cell–ECM interactions. The disadvantages are difficult visualization of 3D structures with automated imaging systems and variations of biological scaffolds from batch-to-batch.	[44,45,46,47,48,49,50,51,52]
Immersion Bioprinting	Cells are mixed with hydrogel and bioprinted (20 μL) into a viscous gelatine bath, layered in 96-well plate. The gelatine bath prevents adhesion of cells to the plate and supports a spherical form of a spheroid. The gelatine bath later on aspirated and substituted with culture medium. The advantage of this method is consistency of spheroids with maintaining a high throughput format.	[53,54]

**Table 2:** Advantages and disadvantages of patient-derived models.

Model	Advantages	Disadvantages
Primary PDC—patient-derived cell culture	– a natural replacement for traditional cancer cell lines – reflection of the characteristics of the patient’s tumor – possibility of high-throughput screening – low cost	– difficult to obtain and subject to aging – not reproducible by 2D culture of tumor heterogeneity and its microenvironment
PDS—patient-derived spheroids	– reflection of the three-dimensional architecture of the tumor – establishment of a suitable large-scale screening – low cost	– do not reflect the cellular composition and microenvironment of the tumors
PDO—patient-derived organoids	– ability to self-assemble on a scaffold that mimics the extracellular environment – ability to differentiate, forming a complex tissue structure – repetition of the structure of the primary tissue – fast distribution and suitable for high bandwidth systems	– the absence of a native tumor microenvironment, which significantly affects the survival of tumor cells
PDTSC—patient-derived tissue slice culture	– preservation of metabolic activity and morphological integrity – preservation of histopathology, tumor microenvironment, including intercellular contacts and a wide range of cells – less time spent due to fewer manipulations (model can be created within a day)	– applicable only to solid neoplasms – do not fully reflect the complexity of the tumor, have a finite life expectancy
Mouse PDX—patient-derived xenografts	– reproduction of complex tumor microenvironment – tumor cell heterogeneity and original tumor architecture – immunomodulation – exposure of tumor cells to a wide range of cytokines, chemokines, growth factors and hormones in vivo – biological similarity between the human disease and the animal model	– limited engraftment success rates, graft rejection and prolonged engraftment, requiring at least 6–7 weeks for engraftment – long time for experiments – high cost
Zebrafish PDX	– quick establishing – rapid turnaround – low-cost – high-throughput – transparency—easy to monitor non-invasively – small space requirements – low doses of screened drugs	– difficult translatability/applicability of dosing results; – different body temperatures between fish and humans; – short observation period; – high mortality
CAM-PDX	– quick establishing – easy to manipulate – high successful transplantation rate – low-cost – high-throughput – chick embryos are naturally immunodeficient; – small space requirements; – low doses of screened drugs	– short observation period; – difference in drug metabolism and immune system with mammals
